# NDRG1 enhances the sensitivity of cetuximab by modulating EGFR trafficking in colorectal cancer

**DOI:** 10.1038/s41388-021-01962-8

**Published:** 2021-08-12

**Authors:** Guang Yang, Ling Huang, Hongtao Jia, Batuer Aikemu, Sen Zhang, Yanfei Shao, Hiju Hong, Galiya Yesseyeva, Chenxing Wang, Shuchun Li, Jing Sun, Minhua Zheng, Junjun Ma

**Affiliations:** 1 Department of General Surgery, Ruijin Hospital, Shanghai Jiao Tong University School of Medicine, Shanghai, China; 2 Shanghai Minimally Invasive Surgery Center, Ruijin Hospital, Shanghai Jiao Tong University School of Medicine, Shanghai, China

**Keywords:** Colorectal cancer, Predictive markers

## Abstract

N-myc downstream-regulated gene 1 (NDRG1) is a key regulator that interacts with many classic tumor signaling pathways, including some molecules downstream of the epidermal growth factor receptor (EGFR). However, whether NDRG1 is involved in the mechanism of resistance to cetuximab (CTX), the first monoclonal antibody targeting the EGFR has not been reported. Here, we found that NDRG1 enhanced the sensitivity of CTX in colorectal cancer (CRC) cell lines. Afterwards, we determined the underlying mechanism of this phenomenon. We demonstrated that NDRG1 inhibited the expression of EGFR; blocked EGFR phosphorylation and reduced the EGFR distribution in the cell membrane, cytoplasm and nucleus. And then, NDRG1 suppressed the EGFR downstream signaling: RAS/RAF/ERK and PI3k/AKT/mTOR pathways. Moreover, we discovered that NDRG1 attenuated the endocytosis and degradation of EGFR induced by caveolin-1 (Cav1). Additionally, our findings were further observed in an animal model and human tissues. Our results represent a potentially significant discovery that explains the mechanisms of NDRG1 in CTX resistance. NDRG1 could be a promising biomarker to predict optimum responses to CTX, and a key target to enhance CTX activity in the treatment of metastatic CRC (mCRC).

## Introduction

Colorectal cancer (CRC) is one of the most common malignancies worldwide, with 1,880,725 new cases and 915,880 deaths in 2020, and the increasing incidence has not been reversed in recent years [1, 2]. Despite immense efforts to promote screening strategies, a tremendous number of CRC patients are diagnosed at an advanced stage.

Several clinical trials have demonstrated that patients with unresectable metastatic colorectal cancer (mCRC) benefit from targeted therapy, particularly the anti-epidermal growth factor receptor (EGFR) antibody, cetuximab (CTX), which has been reported to significantly improve progression-free survival (PFS) and overall survival (OS) within the RAS/BRAF wild-type (wt) mCRC population [3]. CTX, which is currently approved to treat mCRC patients with RAS wt tumors due to the insensitivity of RAS mutation tumors to CTX, has been included in the National Comprehensive Cancer Network and European Society for Medical Oncology guidelines based on the results of many clinical trials [4–6]. Among these trials, the CALGB/SWOG 80405 trial reported that in KRAS wt tumors, the response rate to CTX treatment was 59.6%. The FIRE-3 trial indicated that among the FOLFIRI plus CTX group, the objective response rate was 65% in the KRAS wt subgroup and 13% in the KRAS mutation subgroup. However, more in-depth analysis of these data revealed that KRAS wt CRC patients were not fully sensitive to CTX treatment; furthermore, there were still CTX-sensitive patients within the KRAS mutation group. This suggests that in addition to RAS gene status, other molecular mechanisms may be involved in the process of the primary and acquired resistance to CTX. Therefore, it is necessary to further identify more precise biomarkers to evaluate CTX sensitivity to improve the therapeutic effect of individualized and comprehensive mCRC treatment.

N-myc downstream-regulated gene 1 (NDRG1), the first of several NDRG family members to be discovered, encodes a 43-KD protein that can be activated by iron chelators [7]. Many recent studies have demonstrated that NDRG1 is closely associated with the development and progression of solid tumors, especially their malignant invasion and metastasis [8, 9], and participates in the regulation of some classical signaling pathways, such as the Ras/Raf/ERK and PI3K/Akt/mTOR pathway [10, 11], which are also the downstream targets of EGFR. However, the effect of NDRG1 on EGFR targeted drug susceptibility has not yet been reported. In addition, our previous study confirmed that NDRG1 could promote Cav1 ubiquitylation in CRC cells [12], and Cav1-mediated endocytosis was found to directly interact with diverse molecules related to extracellular protein trafficking and signaling transduction, including EGFR [13]. Naturally, we wondered whether NDRG1 affects EGFR endocytosis by inhibiting Cav1, thus modifying the sensitivity of CRC cells to CTX.

In this study, for the first time, we have shown that NDRG1 could enhance the sensitivity of CRC to CTX. Furthermore, we revealed its underlying mechanism through (1) the regulation of EGFR expression, distribution, and phosphorylation and (2) the regulation of EGFR endocytosis and degradation. This study may provide a theoretical basis for finding new biomarkers to evaluate of individualized treatment for mCRC.

## Results

### NDRG1 enhanced CTX sensitivity in CRC cell lines

**Fig. 1:**
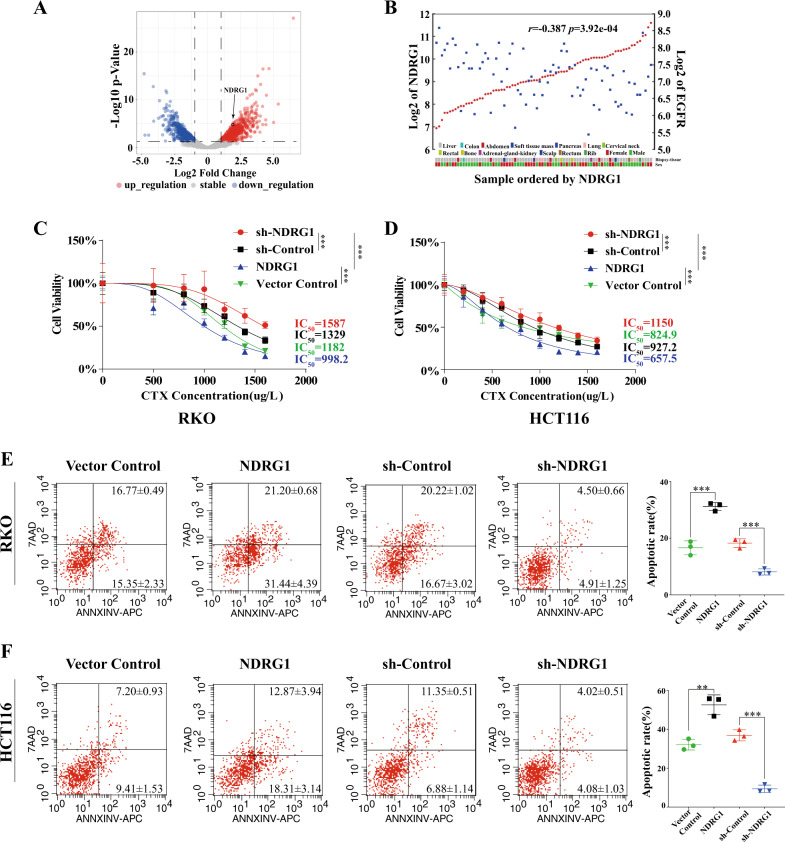
NDRG1 enhanced CTX sensitivity in CRC cell lines. **A** Analyses from the NCBI-GEO dataset exhibited the variation of NDRG1 level in CTX-resistant CRC cells. **B** Analyses from the NCBI-GEO dataset revealed the correlation of the mRNA expression level between NDRG1 and EGFR in CRC samples (Pearson’s correlations). **C**, **D** NDRG1-overexpression and NDRG1-knockdown RKO and HCT116 cells were treated with increasing concentrations of CTX (200–1600 μg/l) for 72 h with 1% FBS. Cell viability was determined by the CCK-8 assay. (*n* = 6, two-way ANOVA). **E**, **F** Fluorescence activated cell sorting (FACS) showing the apoptotic rate of cells stained with Annexin V-APC/7AAD following 48 h of treatment with CTX (1000 μg/l) (*n* = 3, Student’s *t* test). The quantitative data were presented as mean ± SD. Error bar represented at least three independent experiments. (NS no significant, *p < 0.05, **p < 0.01, ***p < 0.001).

To identify the effect of NDRG1 on the response of CRC cells to CTX, we first used data from the public GEO database. By analyzing primary data from dataset GSE71210, in which Affymetrix HG-U133A array was used to compare the expression patterns of genes between parental DiFi CRC cells and CTX-resistant (CR) DiFi5 cells, the result showed that NDRG1 was obviously overexpressed in the CR cells (1.89-fold increase, *p* < 0.001) (Fig. 1A). In another database GSE5851, a significant negative correlation between NDRG1 and EGFR mRNA expression levels was observed in 80 CRC samples which received CTX treatment (*r* = −0.381, *p* < 0.001) (Fig. 1B). In addition, our team previously reported that the level of the phosphorylated NDRG1 protein (NDRG1-pT346) is significantly associated with EGFR, EGFR-pY1068, and EGFR-pY1173 protein expression in gastric cancer [14]. These results illustrated that NDRG1 might be involved in regulating the expression of EGFR and then affecting the sensitivity to CTX.

Next, we used the RKO and HCT116 cell lines to establish the stable NDRG1-overexpression and NDRG1-knockdown clones, and then tested their sensitivity to CTX by CCK-8 assay. In both the RKO and HCT116 cell lines, the IC_50_ for CTX with NDRG1-overexpression was significantly decreased compared with that in the control group (RKO: 998.2 μg/ml vs 1182 μg/ml, *p* < 0.001; HCT116: 657.5 μg/ml vs 824.9 μg/ml, *p* < 0.001). In contrast, a major difference in IC_50_ values in response to CTX was observed between NDRG1-knockdown and control cells (RKO: 1587 μg/ml vs 1329 μg/ml, *p* < 0.001; HCT116: 1150 μg/ml vs 927.2 μg/ml, *p* < 0.001) (Fig. 1C, D). Furthermore, an apoptosis assay revealed that when both cell lines were challenged with CTX, NDRG1-overexpressing cells showed a significantly increased apoptotic rate relative to that of the control group, and NDRG1-knockdown cells were more insensitive to CTX than control cells (Fig. 1E, F). These data revealed that the variation in NDRG1 levels had an obvious effect on the sensitivity of CRC cells to CTX.

### NDRG1 reduced EGFR protein expression in CRC cell lines

**Fig. 2:**
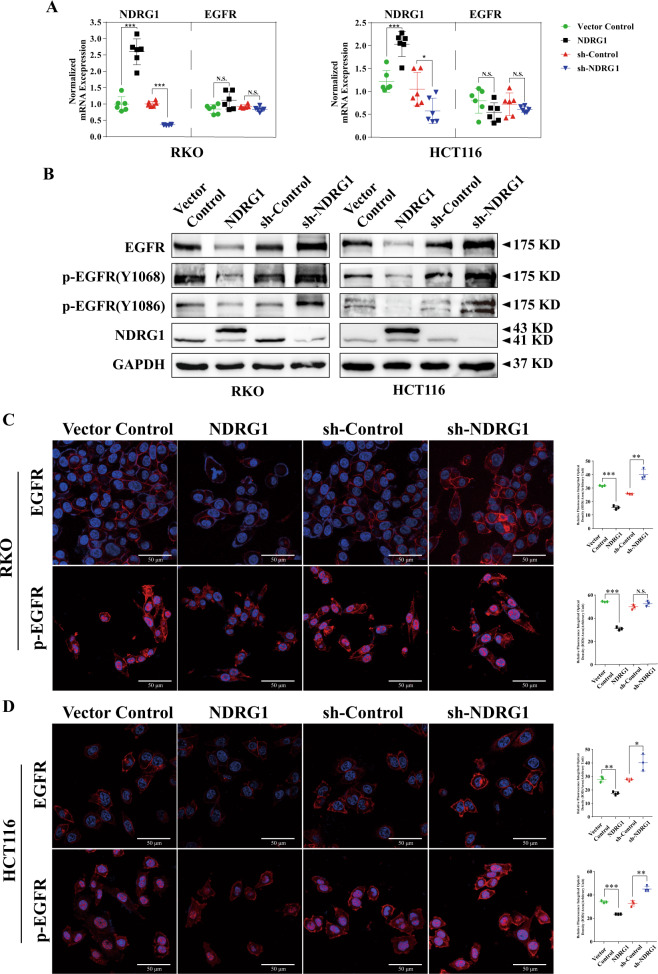
NDRG1 reduces EGFR protein expression in CRC cell lines. **A** qPCR results of the NDRG1 and EGFR mRNA expressions in NDRG1-overexpression and NDRG1-knockdown RKO and HCT116 cells compared with their relative control cells (*n* = 3, Student’s *t* test). **B** Western blots for EGFR and p-EGFR proteins in NDRG1-overexpression and NDRG1-knockdown RKO and HCT116 cells compared with their relative control cells. **C**, **D** Representative images of EGFR and p-EGFR expression by immunofluorescence staining. Scale bar = 50 μm. The quantitative data were presented as mean ± SD. Error bar represented at least three independent experiments. (NS no significant, *p < 0.05, **p < 0.01, ***p < 0.001).

After confirming that NDRG1 could impact CTX sensitivity, we aimed to reveal the underlying mechanisms of this phenomenon. CTX specifically binds with EGFR expressed on the tumor cell membrane and competitively blocks EGF and other ligands, subsequently inhibiting downstream signals. Therefore, EGFR is the primary regulator of CTX sensitivity. First, qPCR was conducted to elucidate whether NDRG1 could regulate EGFR at the mRNA level. In comparison to the corresponding control groups, neither the NDRG1-overexpressing or NDRG1-silenced subsets of RKO or HCT116 cells showed a significant change in EGFR mRNA expression (Fig. 2A). Then, immunoblotting was performed to investigate the relationship between NDRG1 and EGFR, phosphorylated EGFR (p-EGFR) at the protein level. EGFR and p-EGFR at Y1068 and Y1086 expression was negatively associated with NDRG1 expression in both cell lines (Fig. 2B).

The expression of EGFR and p-EGFR in both cell lines was further investigated via immunofluorescence (IF). When NDRG1 was exogenously overexpressed, the level of these two proteins at the cell membrane was remarkably decreased. Conversely, in NDRG1-silenced RKO and HCT116 cells, larger amounts of EGFR accumulated at the membrane comparing with those in the control cells and the expression of p-EGFR also increased in HCT116 knockdown cells. While in RKO cells, NDRG1-knockdown did not have an evident effect on the p-EGFR expression (Fig. 2C, D and Supplementary Fig. 1a–d). Detection of the expression of EGFR and p-EGFR on cell membrane by flow cytometry was also verified the tendency in NDRG1 overexpressed and knocked down cells, respectively (Supplementary Fig. 1e). These data suggested that NDRG1 did not regulate EGFR at the mRNA level but inhibited its protein expression, especially on the cell membrane.

### NDRG1 inhibited the phosphorylation of EGFR and downstream signaling molecules

Autophosphorylation of EGFR, the primary transformation that occurs after ligand induction, is a key characteristic of EGFR activation and signaling [15]. EGFR phosphorylation at Y1068 and Y1086 was examined in the current study and found to be directly involved in activating the RAS and PI3K pathways [16]. After overnight starvation, the cells were simulated by EGF (10 ng/ml) and immunoblotting was repeated, which showed that the tendency of NDRG1 to inhibit expression of p-EGFR at Y1068 and Y1086 was unchanged (data not shown).

**Fig. 3:**
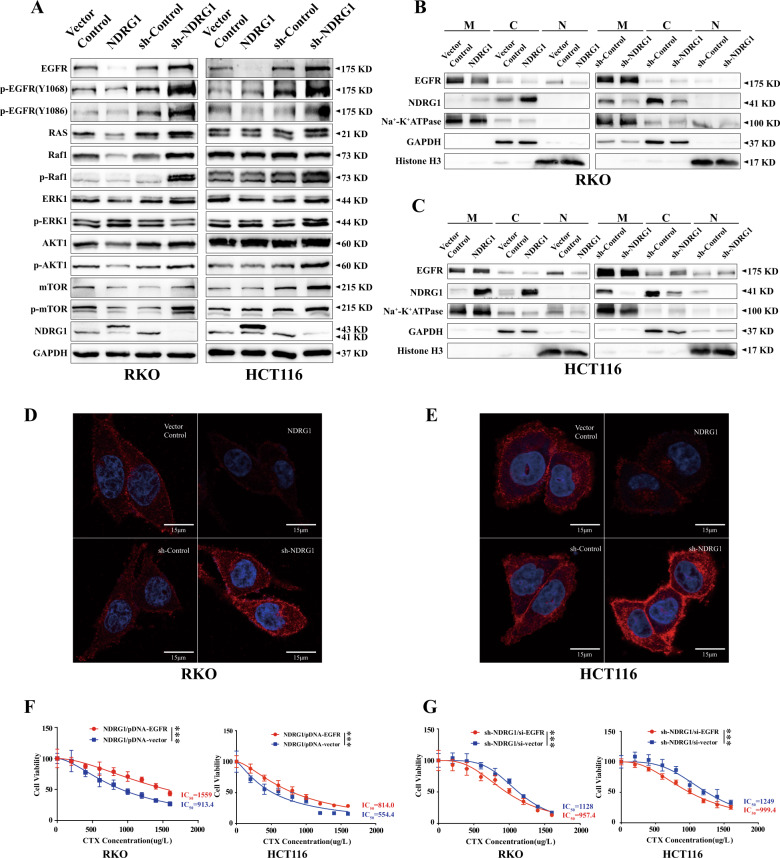
NDRG1 affects the sensitivity of CTX in CRC cells through regulating the EGFR expression, phosphorylation, and distribution. **A** Western blots for proteins of RAS/RAF/ERK and PI3k/AKT/mTOR pathways in NDRG1-overexpression and NDRG1-knockdown RKO and HCT116 cells compared with their relative control cells. GAPDH was used as a loading control. **B**, **C** The protein located on the membrane, cytoplasm, and nucleus were isolated by subcellular fractionation assay after NDRG1 was overexpressed or silenced, respectively, and then the variation of EGFR was detected by western blot. Na^+^-K^+^ATPase, GAPDH and Histone H3 were used as loading control for the membrane (M), cytoplasm (C), and nucleus (N), respectively. **D**, **E** Representative images of EGFR distribution on cells by immunofluorescence staining. Scale bar = 15 μm. **F** NDRG1-overexpression cells were transfected with EGFR-overexpression plasmid and vector plasmid, respectively, and then the sensitivity to CTX was detected by CCK-8 assay (*n* = 6, two-way ANOVA). **G** NDRG1-knockdown cells were transfected with EGFR-siRNA and vector siRNA, respectively, and then the sensitivity to CTX was detected by CCK-8 assay (*n* = 6, two-way ANOVA). The quantitative data were presented as mean ± SD. Error bar represented at least three independent experiments. (NS no significant, *p < 0.05, **p < 0.01, ***p < 0.001).

We further detected the activation of representative markers involved in the RAS and PI3K pathways by the two proteins above. In both cell lines, RAS, p-Raf1, ERK1, p-Akt1, mTOR, and p-mTOR were found to be negatively regulated by NDRG1. Only the expression of the p-ERK1 protein in RKO cells was positively regulated by NDRG1 and the expression of Raf1 and Akt1 was not affected in HCT116 cells (Fig. 3A and Supplementary Fig. 2a).

Together, these data demonstrated that in both RKO and HCT116 cells, NDRG1 not only suppressed total EGFR protein expression, but also inhibited EGFR phosphorylation at Y1068 and Y1086. In addition, even though the modulatory effect of NDRG1 were not exactly the same in two cells, NDRG1 had an inhibitory effect on downstream signaling pathways.

### NDRG1 inhibited the EGFR distribution in subcellular organelles

To better define whether NDRG1 impacts the EGFR distribution, we performed subcellular fractionation analysis to examine the expression of this protein in the membrane, cytoplasm, and nucleus (Fig. 3B, C). The results showed a slightly reduction in EGFR expression in these subcellular organelles after NDRG1 was overexpressed in both RKO and HCT116 cells. When NDRG1 was silenced, the expression of EGFR in the membrane and cytoplasm in HCT116 cells was increased. In addition, obviously accumulation of EGFR in the membrane, cytoplasm and nucleus were observed in both NDRG1-knockdown cells from the confocal photograph (Fig. 3D, E). When combined, these results suggest that NDRG1 inhibits the distribution of EGFR in subcellular structures in some CRC cell lines.

### NDRG1 affected the sensitivity of CRC cells to CTX by regulating EGFR expression

To further verify the mechanism by which NDRG1 enhanced sensitivity to CTX by regulating EGFR expression, EGFR-overexpression plasmids and EGFR-siRNA as well as the corresponding negative controls were transfected into NDRG1-overexpression and NDRG1-knockdown cells, respectively, after which the drug sensitivity was tested by CCK-8 assay. The transfection efficiency was testified by immunoblotting (Supplementary Fig. 3a, b). Then, in comparison to the negative control cells, the NDRG1-overexpression cells after EGFR-overexpression plasmids transfection turned to insensitive to CTX (IC_50_: 1559 μg/ml vs 913.4 μg/ml in RKO cells, *p* < 0.001 and 814.0 μg/ml vs 554.4 μg/ml in HCT116 cells, *p* < 0.001) (Fig. 3F). And the transfection of EGFR-siRNA restored the change in CTX resistance induced by downregulating NDRG1 (IC_50_: 957.4 μg/ml vs 1128 μg/ml in RKO cells, *p* < 0.001 and 999.4 μg/ml vs 1249 in HCT116 cells, *p* < 0.001) (Fig. 3G).

Taken together, these results suggested that NDRG1 increased CTX sensitivity by suppressing EGFR expression in RKO and HCT116 cells.

### Knockdown of NDRG1 markedly accelerated the endocytosis of EGFR

**Fig. 4:**
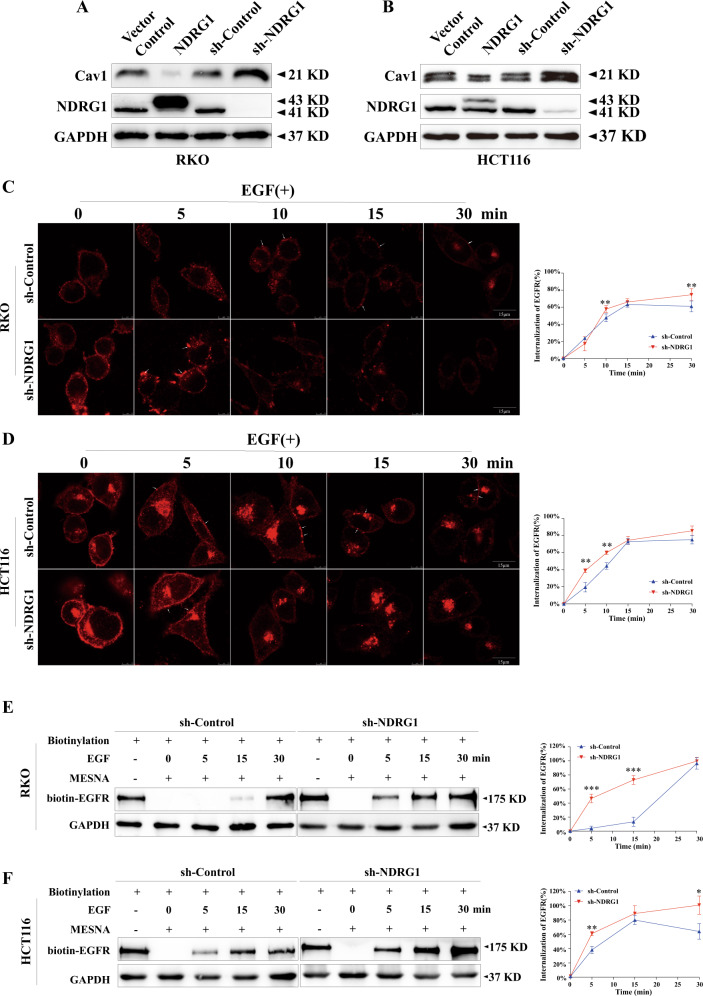
Knockdown of NDRG1 markedly accelerates the endocytosis of EGFR. NDRG1-knockdown and the corresponding control cells were treated with EGF (50 ng/ml) for 5–30 min after starvation overnight. **A**, **B** Western blots for Cav1 proteins in NDRG1-overexpression and NDRG-knockdown RKO and HCT116 cells compared with the corresponding control cells. **C**, **D** The endocytosis of EGFR (red) was determined by indirect immunofluorescence (white arrow: the EGFR accumulated on cell membrane). Data were quantified as described in “Materials and methods”. (*n* = 6, Student’s *t* test). Scale bar = 15 μm. **E**, **F** The endocytosis of EGFR was determined by cell surface biotinylation as described in “Materials and methods” (*n* = 3, Student’s *t* test). Control: total biotinylated proteins without incubation with MESNA to remove biotin from the sulfo-NHS-SS-biotin-labeled proteins on the cell surface and without stimulation with EGF. The quantitative data were presented as mean ± SD. Error bar represented at least three independent experiments. (NS no significant, *p < 0.05, **p < 0.01, ***p < 0.001).

First, western blotting was performed to test the relationship between NDRG1 and Cav1 in RKO and HCT116 cells. The results indicated that NDRG1 negatively regulated the expression of Cav1 in clones in which NDRG1 was either overexpressed or knocked down (Fig. 4A, B). Then, IF was conducted to observe the process of EGFR endocytosis in NDRG1-silenced cells and the negative control. After stimulation with EGF (50 ng/ml), NDRG1-silenced RKO cells started to endocytose a small amount of EGFR. After 5 min, EGFR internalization was obviously accelerated, and exceeded that of the control cells until 30 min after stimulation (Fig. 4C and Supplementary Fig. 4a, b). In HCT116 cells, EGFR endocytosis in NDRG1-silenced cells was accelerated relative to that in the control group from the beginning of the experiment (Fig. 4D and Supplementary Fig. 4c, d).

Surface biotinylation experiment was conducted to further confirm this process. Upon EGF stimulation, EGFR was strongly internalized for 0–30 min in NDRG1-knockdown RKO cells, but EGFR endocytosis was greatly delayed in control cells by 15 min (Fig. 4E). In HCT116 cells, the rate of EGFR internalization of the control group was significantly lower than that of NDRG1-knockdown group and had even decreased after 15 min (Fig. 4F). This phenomenon might be accounted for by the recycling of already present EGFR at that time.

Overall, these results agreed with our previous assumption that the inhibition of NDRG1 expression would increase the extent and rate of EGFR endocytosis.

### Knockdown of NDRG1 promoted the endocytosis of EGFR by upregulating Cav1

**Fig. 5:**
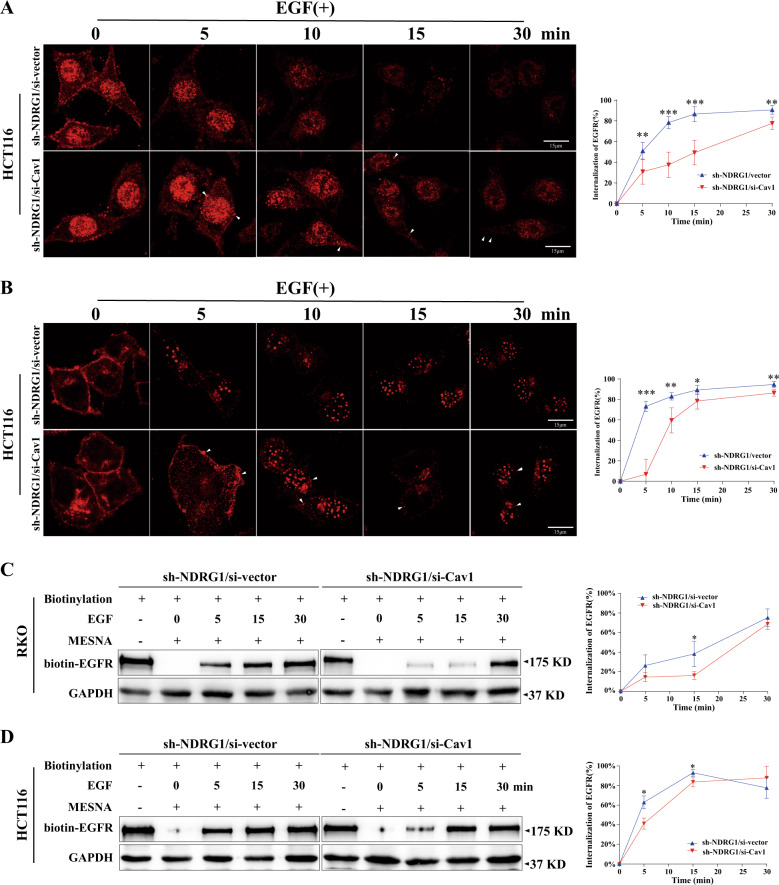
The inhibition of Cav1 retarded the endocytosis of EGFR and enhanced the sensitivity in NDRG1-knockdown cells. NDRG1-knockdown cells were transfected with siRNA-Cav1 and vector siRNA, respectively. **A**, **B** The endocytosis of EGFR (red) was determined by indirect immunofluorescence (white arrow: the EGFR accumulated on cell membrane). Data were quantified as described in “Materials and methods”. (*n* = 6, Student’s *t* test). Scale bar = 15 μm. **C**, **D** Endocytosis of EGFR by cell surface biotinylation (*n* = 3, Student’s *t* test). The quantitative data were presented as mean ± SD. Error bar represented at least three independent experiments. (NS no significant, *p < 0.05, **p < 0.01, ***p < 0.001).

Several pathways have so far been confirmed to mediate the endocytosis of EGFR [17–19]. To demonstrate the impact of NDRG1 on EGFR endocytosis through its interaction with Cav1, Cav1-siRNA and the vector were transfected into NDRG1-knockdown RKO and HCT116 knockdown cells (Supplementary Fig. 3c). Through IF imaging and quantification of the data, EGFR endocytosis in both NDRG1-knockdown cells was significantly delayed after Cav1-siRNA transfection compared with that in the control cells (Fig. 5A, B and Supplementary Fig. 5a, b). Consistent with our IF results, biotinylation assay also showed that the endocytic activities in both NDRG1-knockdown cell lines transfected with Cav1-siRNA following the addition of EGF were significantly inhibited relative to those in control cells (Fig. 5C, D and Supplementary Fig. 5c, d).

Taken together, these results suggested that Cav1 played an important role in increasing EGFR endocytosis in NDRG1-knockdown cells.

### Knockdown of NDRG1 promoted the degradation of EGFR by upregulating Cav1

**Fig. 6:**
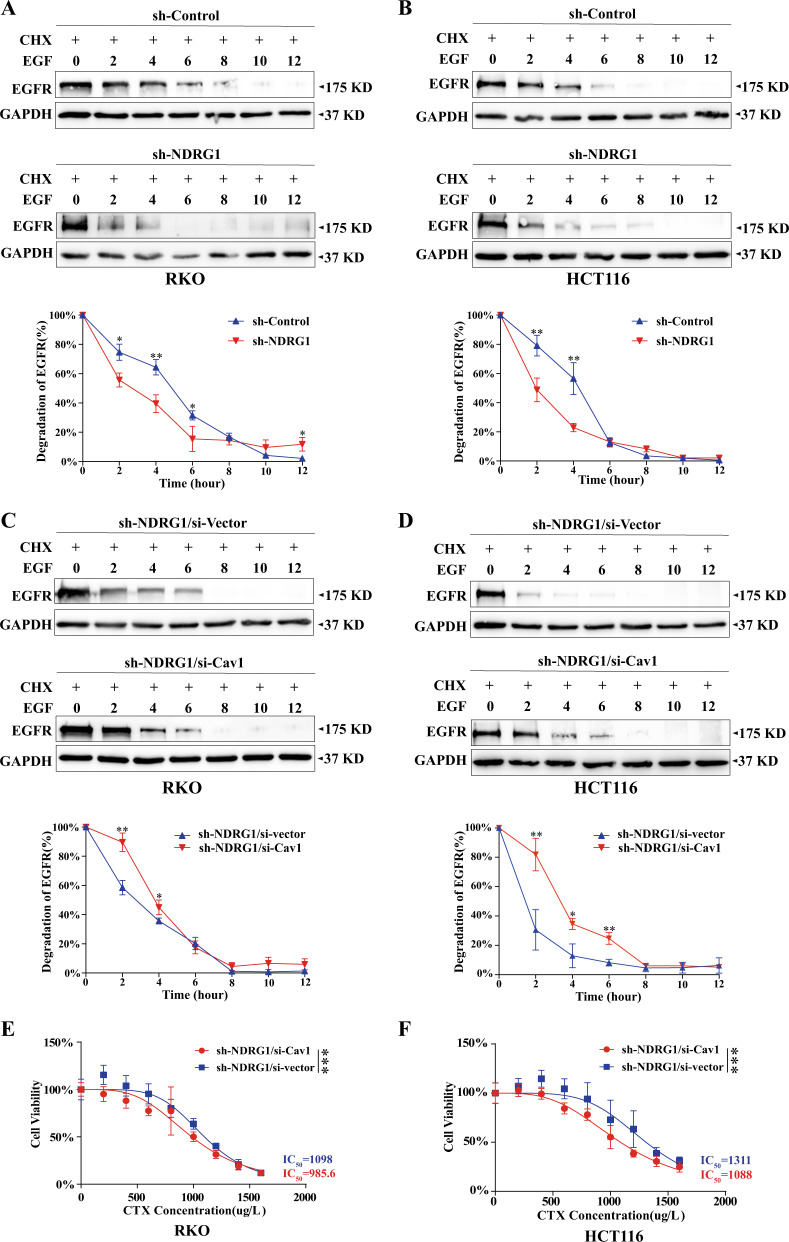
Knockdown of NDRG1 promoted the degradation of EGFR, the inhibition of Cav1 resuced EGFR from degradation and enhanced the sensitivity to CTX in NDRG1-knockdown cells. **A**, **B** The degradation of EGFR in NDRG1-knockdown and the corresponding control cells was determined by western blots as described in “Materials and methods” (*n* = 3, Student’s *t* test). **C**, **D** The degradation of EGFR in NDRG1-knockdown cells after Cav1-siRNA and the vector transfection, respectively (*n* = 3, Student’s *t* test). **E**, **F** The sensitivity to CTX was examined in NDRG1-knockdown cells after Cav1-siRNA and the vector transfection, respectively, by CCK-8 assay (*n* = 6, two-way ANOVA). The quantitative data were presented as mean ± SD. Error bar represented at least three independent experiments. (NS no significant, *p < 0.05, **p < 0.01, ***p < 0.001).

After endocytosis, the modulation of receptor degradation is essential for proper EGFR signaling. Thus, the impact of NDRG1 on EGFR degradation was further explored. NDRG1-knockdown RKO and HCT116 cells and the corresponding control cells were examined to determine the EGFR degradation. In RKO cells, the suppression of NDRG1 expression induced a dramatic increase in EGFR degradation over the first 6 h (degradative rate: 84.5% vs 68.5%, *p* < 0.05) (Fig. 6A), which gradually plateaued and was even slightly slower than that in the control cells. In HCT116 cells, 77.0% of EGFR was degraded after 4 h, compared with the degradation rate of 43.4%, in the relative control cells (Fig. 6B, *p* < 0.05). Next, the destruction of EGFR was re-examined after the transfection of Cav1-siRNA and vector in both cell lines. The degradation of some EGFR was rescued after the inhibition of Cav1 (Fig. 6C, D).

Together, these results illustrated that the inhibition of NDRG1 in CRC cells significantly accelerated the degradation of EGFR, which was internalized into cells through Cav1-mediated endocytosis.

### Interruption of Cav1 in NDRG1-knockdown cells reversed resistance to CTX

The constructed cell clones described above were confronted with sustained challenge with CTX at different concentrations. After Cav1 was suppressed, the IC50 in both cell lines was remarkably declined in comparison with those in negative control cells (985.6 μg/ml vs 1098 μg/ml in RKO cells, *p* < 0.001; and 1088 μg/ml vs 1311 μg/ml in HCT116 cells, *p* < 0.001) (Fig. 6E, F).

### Knockdown of NDRG1 markedly impedes the sensitivity of CTX in vivo

**Fig. 7:**
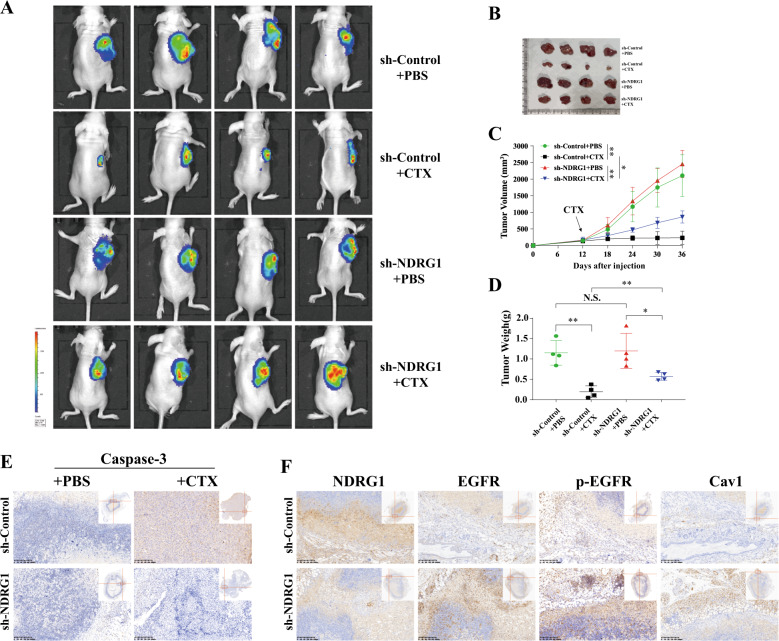
Knockdown of NDRG1 impedes the sensitivity of CTX in vivo. **A** All the mice were imaged when the largest diameter of tumor reached 20 mm after CTX treatment. The color scale depicting the photon fluxes emitted from the tumor cells. **B** Macroscopic views showing the xenografts in the indicated subgroups. **C** Plot representing the evolution of tumor volume of xenografts in the indicated groups (*n* = 4, two-way ANOVA). **D** tumor weight of xenografts in the indicated groups (*n* = 4, Student’s *t* test). **E** The apoptotic cells in the dissected xenografts were immunostained with Caspase-3; magnification: ×10. **F** The representative IHC staining images of NDRG1, EGFR, p-EGFR(Y1068) and Cav1 in tumor tissues; magnification: ×10. The quantitative data were presented as mean ± SD. (NS no significant, *p < 0.05, **p < 0.01, ***p < 0.001).

GFP-luciferase-labeled NDRG1-knockdown cells and the corresponding control cells were injected into mice to establish tumor xenografts. Then, the mice were randomly divided into four groups and treated with CTX or placebo. From the bioluminescence images, we observed that the tumor xenografts grew remarkably larger in the NDRG1-knockdown group than in the control group (Fig. 7A–C). Although tumor growth was partially limited by CTX in NDRG1-knockdown cells, this tendency of increased growth was not dramatically reversed. In contrast, the control group treated with the drug was more sensitive to CTX, as indicated by the arrest of tumor growth over the whole experimental period (Fig. 7A–C). Similarly, after treatment, the average tumor weight of the control group was significantly smaller than that of the NDRG1-knockdown group (196 ± 145 mg vs 573 ± 96 mg, *p* < 0.01) (Fig. 7D). In addition, IHC analysis of these samples was also conducted to examine cell apoptosis (caspase-3 staining), and detect NDRG1, EGFR, p-EGFR, and Cav1. The images showed that CTX enhanced cell apoptosis in the control group (Fig. 7E). Obvious and deep staining for EGFR, p-EGFR, and Cav1 was observed in NDRG1-knockdown tumors, but the staining was weaker in control tumors (Fig. 7F).

### NDRG1 expression is negatively related with EGFR expression and promoted the sensitivity of human CRC tissues to CTX

**Fig. 8:**
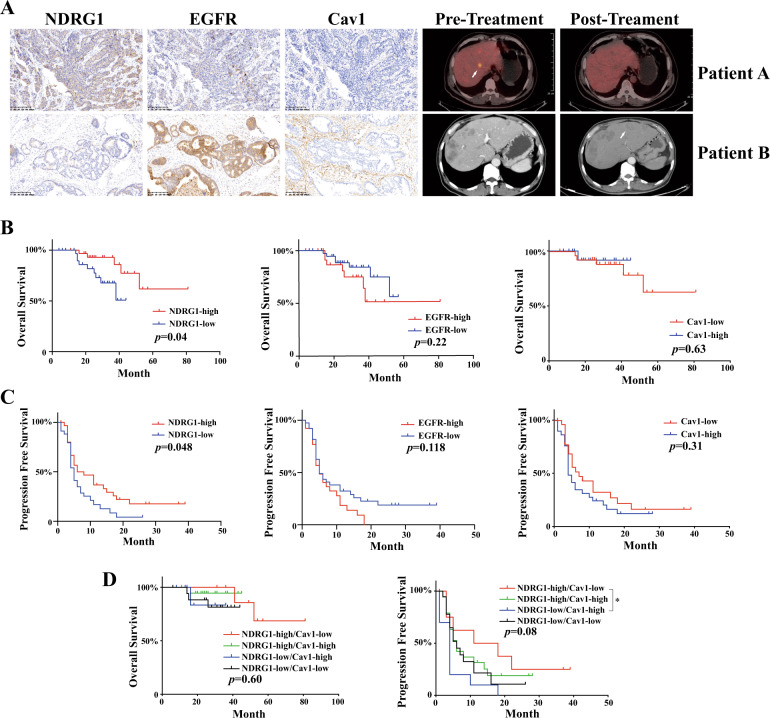
Clinical significance of the NDRG1/EGFR pathway in CRC patients with CTX treatment. **A** Representative IHC images of two CRC cases showed the negative correlation between NDRG1 and EGFR, Cav1. Image examination contrasted the reaction of CTX treatment in these two cases (white arrow: liver metastases). **B** OS of the colorectal cancer patients with NDRG1, EGFR, and Cav1 expression. (Log-rank test). **C** PFS of the colorectal cancer patients with NDRG1, EGFR, and Cav1 expression. (Log-rank test). **D** OS and PFS curves of the colorectal cancer patients which stratified by NDRG1 and Cav1. The quantitative data were presented as mean ± SD. (NS no significant, *p < 0.05, **p < 0.01, ***p < 0.001).

Determination of the expression of NDRG1, EGFR, and Cav1 by IHC assay showed a significant inverse correlation between NDRG1 and EGFR (*R* = −0.528, *p* < 0.001) and NDRG1 and Cav1 (*R* = −0.322, *p* = 0.016) (Fig. 8A and Table 1). Nearly half of the patients (32/65) were rectal cancer cases, whereas the location was not associated with NDRG1 expression. Among the high NDRG1 expression group, 61.3% (19/31) of cases showed sensitivity to CTX treatment, whereas only 35.3% (12/34) cases in the low NDRG1 expression group (*R* = −0.280, *p* = 0.023). Beyond that, EGFR and Cav1 were failed to predict the sensitivity to CTX (Table 1).

**Table 1:** The relationship between NDRG1 expression and EGFR, Cav1, the sensitivity to CTX in this study.

Varibale	*N*	NDRG1		*p* value	*N*	Response to CTX		*p* value
		High-expression	Low-expression			Sensitive	Resistant	
Age				0.325				0.325
≤60	39	20	19		39	20	19	
>60	26	11	15		26	11	15	
Sex				0.146				0.017
Male	43	18	25		43	16	27	
Female	22	13	9		22	15	7	
Tumor location				0.054				0.361
Colon	33	12	21		34	15	19	
Rectum	32	19	13		31	16	15	
EGFR				<0.001				0.168
High-expression	26	4	22		26	10	16	
Low-expression	39	27	12		39	21	18	
Cav1	56^a^			0.016	56^a^			0.391
High-expression	29	19	10		29	15	14	
Low-expression	27	9	18		27	12	15	
Response to CTX				0.032				
Sensitive	31	19	12					
Resistant	34	12	22					

^a^Nine sample tissues were unsufficient to perform IHC.

In addition, Kaplan–Meier analysis revealed that patients in the low NDRG1 expression group had significantly poor OS than those in the high NDRG1 expression group (Fig. 8B). Moreover, high NDRG1 expression was associated with a longer PFS (Fig. 8C). These meaningful differences were not observed when the samples were divided into groups according to EGFR and Cav1 expression (Fig. 8B, C). Further analysis with a combination of NDRG1 and Cav1 showed there was not obvious difference in OS and PFS between each group, but subgroup analyses revealed that compared with NDRG1-low/Cav1-high group, NDRG1-high/Cav1-low group tended to have a longer PFS (*p* = 0.025) (Fig. 8D). These findings suggested that patients with high NDRG1 levels could benefit from CTX treatment, and that NDRG1 combined with Cav1 might function as an indicator to evaluate CTX sensitivity.

## Discussion

CTX (Erbitux^TM^) has presented evident antitumor activity in clinical treatment, particularly in the mCRC cases [4, 20]. However, similar to other antitumor drugs, CTX is subject to both intrinsic and acquired resistance. Therefore, understanding the molecular mechanisms of CTX resistance is urgent and will undoubtedly improve the effectiveness of this therapeutic drug. In this study, for the first time, we found that NDRG1 can affect the sensitivity to CTX by modulating many processes in EGFR trafficking.

The current most notable predictive biomarker of the response to CTX is the mutational status of the KRAS gene [21]. Therefore, the RAS wt and RAS mutant cell lines RKO and HCT116, respectively, were chosen to examine their sensitivity to CTX. The results showed that the NDRG1 enhanced the sensitivity of both cell lines to CTX. It indicated that regardless of KRAS, NDRG1 is involved in a novel mechanism at the foundation of CTX responsiveness. Interestingly, this result was not consistent with previous bioinformatic data (Fig. 1A). Combined with the result that NDRG1 could not affect the expression of EGFR at mRNA level (Fig. 2A), we speculated that NDRG1 may regulate EGFR protein translation and/or post-translational modifications and consequently alter the sensitivity of CRC cells to CTX.

CTX competitively blocks natural ligand binding, prevents EGFR activation and self-phosphorylation, and suppresses downstream signaling [22, 23]. Thus, the revision of these processes may cause resistance. Our study demonstrated that NDRG1 significantly suppressed both the levels and membrane localization of the receptor in CRC cells. Additionally, NDRG1 remarkably inhibited the levels of EGFR phosphorylated at the Y1068 and Y1086 sites with or without EGF incubation. Moreover, NDRG1 inhibited the two RAS/RAF/ERK and PI3K/AKT/mTOR axes, which are signal downstream of EGFR that play an important role in CTX resistance [24].

In addition to plasma membrane EGFR signaling, the abnormal subcellular localization of EGFR was identified as a mechanism involved in the resistance to CTX. Studies have demonstrated that CTX treatment can promote the nuclear localization of EGFR, whereas elevated levels of nuclear EGFR were found in CTX-resistance non-small-cell lung cancer cells [25]. Our study illustrated that NDRG1 could significantly reduce EGFR localized in membrane, cytoplasm, and nucleus. This result implied that NDRG1 might be involved in the process of EGFR transportation into the nucleus and further affect sensitivity to anti-EGFR drugs.

Additionally, although most EGFR signaling is thought to occur at the plasma membrane [26, 27], many studies have proved that endosomes are involved in EGFR-mediated signals, suggesting the presence of distinct pathways requiring EGFR endocytosis [28, 29]. Moreover, one major destination for EGFR trafficked after internalization is the lysosomal degradation. Sorting in this process is fundamental to the regulation of EGFR signaling as well. Menezes et al. reported that NDRG1 could inhibit EGFR and facilitate its lysosomal processing and degradation by increasing the levels of mitogen-inducible gene 6 [30]. Interestingly, our result suggested that NDRG1-knockdown promoted the internalization and degradation of EGFR in CRC cells which seemingly contradicted our previous results and was not consistent with the study above. The EGFR internalization is a complex process that involves many mechanisms, and different routes have different effects on EGFR fate and signaling [31, 32]. At all physiological EGF concentrations, the EGFR is internalized mainly through clathrin-mediated endocytosis (CME) [33], but clathrin-independent endocytosis including raft/caveolar endocytosis (RCE) also plays important roles, especially at high EGF doses (>10 ng/ml) [34]. The outcome for EGFR trafficking is dependent on the ratio of CME to RCE. We speculated that NDRG1-knockdown promoted the accumulation of Cav1, especially on the cell membrane, which was also confirmed by the colocalization of EGFR and Cav1 (Supplementary Fig. 4a–d). And then high concentration of EGF induced accelerating the internalization of EGFR by RCE.

Furthermore, evidence suggests that the dysregulation of EGFR internalization and degradation contributes to CTX resistance [35, 36]. Our results demonstrated the inhibition of Cav1 obviously decreased these effects and restored the sensitivity of NDRG1-knockdown cells to CTX. Therefore, based on our previous research [12], for the first time, we propose that NDRG1 attenuates EGFR internalization and degradation by promoting Cav1 ubiquitylation, and further enhances the sensitivity to CTX in CRC cells.

NDRG1 enhanced sensitivity to CTX and it was found to be negatively regulated with the expression of EGFR and Cav1 in an animal model and human tissues as well. We also found other than EGFR and Cav1, the level of NDRG1 expression was a better predictor of the prognosis and effectiveness of CTX treatment. However, the *p* value approached 0.05, indicating that the function of NDRG1 needs to be further confirmed in larger CRC sample, and combined with other molecular markers may improve its clinical significance. In addition, together with NDRG1 and Cav1 for the prediction of PFS is limited. More clinical trials need to be conducted to confirm their function. Another deficiency of the study is that only the KRAS wt patients were enrolled and most patients received the combined medication due to the current indications of CTX use. The influence made by other antitumor medicines could not be excluded. The function of NDRG1 in patients with KRAS mutation remains to be further authenticated.

In conclusion, our results demonstrated that NDRG1 enhances the sensitivity to CTX by inhibiting EGFR expression, phosphorylation, distribution, endocytosis, degradation, and downstream signaling (Supplementary Fig. 3b). Our results help to fulfill the mechanisms of CTX resistance and NDRG1 could be a potential biomarker to predict optimum responses to CTX in the treatment of metastatic CRC.

## Material and methods

### Antibodies and regents

CTX was purchased from Merck (Darmstadt, Germany). The primary and secondary antibodies were listed in Supplementary material and methods file.

### Cell culture and transfection

The RKO and HCT116 human CRC cell lines were purchased from the American Type Culture Collection, and both cell lines were authenticated by short tandem repeat profiling. Stable NDRG1-overexpression and knockdown clones were established as described previously [37]. All the cells were cultured in RPMI-1640 medium (Gibco, Grand Island, NY, USA) supplemented with 10% fetal bovine serum (Gibco) and 1% penicillin-streptomycin (New Cell & Molecular Biotech, Suzhou, China) at 37 °C in 5% CO_2_. EGFR-overexpression plasmids and small interfering RNA (siRNA) for EGFR and Cav1 were purchased from GenePharma (Shanghai, China). Transient transfection was performed as previously described [36]. Sequences of siRNA used were showed in the Supplementary file.

### RNA isolation and quantitative real-time PCR (qPCR)

Total RNA isolation and qPCR were performed as described previously [12]. The primer sequences were showed in the Supplementary material and methods file.

### Western blotting, cell viability assay, and apoptosis assay

Immunoblot analysis, cell viability assay, and apoptosis assay were performed as previously described [38]. Subcellular fractionation and protein extraction were carried out by using a subcellular protein fractionation kit according to the manufacturer’s instructions (#78840; Thermo Scientific, Waltham, MA, USA).

### Flow cytometry to detect the protein on cell membrane

Cells were harvested and washed in cold PBS, then incubated in block buffer for 60 min. Next, cells were incubated with primary and second antibodies sequentially for 30 min in the dark. Finally, fully re-suspend the cells by PBS and analyzed by flow cytometry.

### Immunofluorescence and the quantification of receptor endocytosis

Indirect IF microscopy was carried out as described previously [37]. The images were examined and captured using a confocal microscope (Leica TCS SP8, Wetzlar, Germany). The detailed procedures of the quantification were described in the Supplementary material and methods file.

### Cell surface biotinylation assay to examine receptor internalization

A protocol modified from Nishimura and Sasaki was used to examine the internalization of cell surface EGFR [39]. The details were described in the Supplementary material and methods file.

### Ligand-induced EGFR internalization and degradation

To examine the degradation of EGFR, we used a modified protocol from Itziar Pinilla-Macua and Alexander Sorkin [40]. The details were described in the Supplementary material and methods file.

### Clinical characteristics and tissue samples

Specimens taken from a total of 65 patients who received CTX treatment and underwent biopsy or operation were used in this study. Prior patient consent and approval from the Ethics Committee of Ruijin Hospital were obtained. Objective response was evaluated using the modified RECIST criteria [41]. Those cases with complete or partial response, stable disease more than 6 months and acquired resistance were categorized as CTX-sensitive group. The patients were followed up every 3–6 months after treatment.

### Immunohistochemical staining

All the formalin-fixed, paraffin-embedded tumor tissue sections were acquired from the Pathology Department of Ruijin Hospital. Immunohistochemical (IHC) staining was performed according to standard procedures. A semi-quantitative method was utilized to score each slide by two independent pathologists according to the German semi-quantitative scoring system [12, 42], with a score >7 indicating high expression.

### Xenograft model

Twenty four-week-old BALB/c nude female mice (body weight: 16–20 g) were purchased from Vitral River Laboratories (Beijing, China) and housed in a specific pathogen-free environment. A total of 2 × 10^6^ RKO cells including NDRG1-knockdown and relative control cells were subcutaneously injected into the mouse right flank of each mouse to establish tumor xenografts. Once the tumors reached an approximate volume of 150 mm^3^, the mice were randomly divided into four groups with balanced tumor volumes, every group contained four mice. An intraperitoneal injection of PBS or 1.5 mg of CTX/injection was administered every 3 days, and tumor size was measured every 7 days. The treatment was not blind to the investigator. After a tumor diameter of 20 mm was reached, all the mice were sacrificed, and the tumors were collected for further IHC staining. All the experimental procedures had been approved by the Shanghai Medical Experimental Animal Care Commission.

### Statistical analysis

All data are presented as the means ± standard deviations and were calculated with SPSS 22 software and GraphPad Prism 7. Two-sided unpaired Student’s *t* test, the *χ*^2^-test, two-way ANOVA, the Kaplan–Meier method and the log-rank test were used to analyze the data. Differences with a *p* values < 0.05 were considered statistically significant.

## Supplementary information

Supplemental Figure 1

Supplemental Figure 2

Supplemental Figure 3

Supplemental Figure 4

Supplemental Figure 5

Supplemental Figure Legends

Supplemental Material and Methods

## Data Availability

The Gene Expression Omnibus (GEO) accession numbers for the data used in this paper are GSE71210 and GSE5851. The analysis of GSE71210 profiles was performed through R package. The analysis of GSE5851 profiles was performed via the R2 Genomics Analysis and Visualization Platform (http://r2.amc.nl).
