# Supplementary material for: NDRG1 enhances the sensitivity of cetuximab by modulating EGFR trafficking in colorectal cancer
Source: Oncogene. 2021 Aug 12;40(41):5993–6006. doi: 10.1038/s41388-021-01962-8 (PMC8516652; doi:10.1038/s41388-021-01962-8)
Supplement: Supplementary file 6 — Supplemental Figure Legends [file 41388_2021_1962_MOESM6_ESM.doc]

**Supplemental Figure 1** **a, b.** Representative images of EGFR in RKO and HCT116 NDRG1-knockdown cells and the corresponding control cells. **c, d.** Representative images of EGFR in RKO and HCT116 NDRG1-knockdown cells and the corresponding control cells (red: EGFR; blue: DAPI). Scale bar=50 μm. **e.** The expression of EGFR and p-EGFR on RKO and HCT116 cell surface were detected by flow cytometry. The quantitative data were presented as mean ± SD Error bar represented at least three independent experiments. (N.S. no significant, *p < 0.05, **p < 0.01, ***p < 0.001)

**Supplemental Figure 2 a,** Densitometry analyses for EGFR and its downstream signalling in RKO and HCT116 cells. **b,** Representative images of EGFR in RKO and HCT116 NDRG1 cells after NDRG1 was overexpressed and knocked down.(red: EGFR; blue: DAPI). Scale bar=15 μm. The quantitative data were presented as mean ± SD Error bar represented at least three independent experiments. (N.S. no significant, *p < 0.05, **p < 0.01, ***p < 0.001)

**Supplemental Figure 3 a,** The efficiency of EGFR amplification in RKO and HCT116 NDRG1-overexpression cells after EGFR-overexpression plasmid and the corresponding negative control transfection, respectively. **b, c.** the efficiency of EGFR interference in RKO and HCT116 NDRG1-knockdown cells after EGFR-siRNA or Cav-siRNA and the corresponding negative control transfection, respectively. **d.** Schematic diagram for the proposed mechanism underlying NDRG1-enhanced CTX sensitivity.

**Supplemental Figure 4 a,b.** Representative images of EGFR endocytosis in RKO NDRG1-knockdown cells and the corresponding control cells. **c, d.** Representative images of EGFR endocytosis in HCT116 NDRG1-knockdown cells and the corresponding control cells (red: EGFR; green: Cav1; blue: DAPI). Scale bar=15 μm.

**Supplemental Figure 5 a,b.** Representative images of EGFR endocytosis in RKO NDRG1-knockdown cells after Cav-siRNA and the corresponding negative control transfection, respectively. **c, d.** Representative images of EGFR endocytosis in HCT116 NDRG1-knockdown cells after Cav-siRNA and the corresponding negative control transfection, respectively (red: EGFR; green: Cav1; blue: DAPI). Scale bar=15 μm.
