# Supplementary material for: NDRG1 enhances the sensitivity of cetuximab by modulating EGFR trafficking in colorectal cancer
Source: Oncogene. 2021 Aug 12;40(41):5993–6006. doi: 10.1038/s41388-021-01962-8 (PMC8516652; doi:10.1038/s41388-021-01962-8)
Supplement: Supplementary file 7 — Supplemental Material and Methods [file 41388_2021_1962_MOESM7_ESM.doc]

**Supplemental Material and methods**

**Antibodies**

The primary antibodies anti-NDRG1 (#ab124689)，anti-EGFR (#ab52894)，anti-p-EGFR (Y1068) (#ab40815), anti-p-EGFR (Y1086) (#ab32086), anti-Ras (#ab52939), anti-Raf1 (#ab181115), anti-p-Raf1 (#ab173539), anti-ERK1 (#ab32537), anti-p-ERK1 (#ab214168), anti-caveolin-1 (Cav1) (#ab2910), anti-Na^+^-K^+^ATPase (#ab76020), anti-Histone H3 (#ab1791)and the secondary antibodies Alexa Fluor 647 donkey anti-rabbit IgG (#ab150075), Alexa Fluor 555 donkey anti-mouse IgG (#ab150106) were purchased from Abcam (Cambridge, UK); anti-Akt1 (#2938) and anti-p-Akt1 (#9018) were obtained from Cell Signaling Technology (Beverly, MA, USA); anti-mTOR (#sc517464) and anti-p-mTOR (#sc293133) were obtained from Santa Cruz (CA, USA). The primary antibody anti-GAPDH (#60004-1-lg) and the secondary antibodies such as horseradish peroxidase (HRP)-conjugated anti-rabbit and anti-mouse antibodies were purchased from Proteintech (Shanghai, China).

**The sequence of small interfering RNA**

EGFR:

Sense-1: 5’-GUCCGCAAGUGUAAGAAGUTT-3’,

Anti-sense-1: 5’-ACUUCUUACACUUGCGGACTT-3’,

Sense-2: 5’- GCAACAUGUCGAUGGACUUTT-3’,

Anti-sense-2: 5’-AAGUCCAUCGACAUGUUGCTT-3’,

Sense-3 5’-GGAGAUAAGUGAUGGAGAUTT-3’,

Anti-sense-3: 5’-AUCUCCAUCACUUAUCUCCTT-3’.

Cav1:

Sense-1: 5’- GCAUUUGGAAGGCCAGCUUTT-3’,

Anti-sense-1: 5’- AAGCUGGCCUUCCAAAUGCTT-3’,

Sense-2: 5’- GCAGUUGUACCAUGCAUUATT-3’,

Anti-sense-2: 5’- UAAUGCAUGGUACAACUGCTT-3’,

Sense-3: 5’- CCGCAUCAACUUGCAGAAATT-3’,

Anti-sense-3: 5’- UUUCUGCAAGUUGAUGCGGTT-3’.

**The primer sequences**

| Gene | Forward primer | Reverse primer |
| --- | --- | --- |
| NDRG1 | CTGCACCTGTTCATCAATGC | AGAGAAGTGACGCTGGAACC |
| EGFR | CCCACTCATGCTCTACAACCC | TCGCACTTCTTACACTTGCGG |
| GAPDH | TTCAACAGCAACTCCCACTCTT | TGGTCCAGGGTTTCTTACTCC |

**Immunofluorescencec and the quantification of receptor endocytosis**

Specifically, for one cell, two polygons were drawn along the outer and the inner edges of the cell membrane to obtain the whole cell’s integrated density and the interior part integrated density, then the mean grey value of the cell membrane (mean_mem_) was calculated using the image processing software FIJI. The average mean fluorescent intensity of the cell membrane without stimulation by EGF was regarded as the total cell membrane fluorescence intensity (mean_total_). Thus, the percentage of receptor internalized in the cell is was calculated as follows: internalized receptor (percentage) =(1-mean_mem_/mean_total_) x100%.

For each value, at least 6 cells from different visual fields were analyzed. The error bar is the standard error.

**Cell surface biotinylation assay to examine receptor internalization**

Briefly, the cells were starved overnight and then incubated with 0.5 mg/ml sulfo-NHS-SS-biotin (#21331; Thermo Scientific, Waltham, MA, USA) in D-PBS (pH 7.4: 8.06 mM Na_2_HPO_4_, 1.47 mM KH_2_PO_4_) on ice for 2 hours. Then, the cells were washed three times with 50 mM NH_4_Cl on ice for 5 min to quench the free biotin in the D-PBS. The cells were then stimulated with EGF for 0, 5, 15, and 30 min. Then, the cells were incubated with 100 mM 2-mercaptoethanesulfonate (MESNA, #M1511; Merck, Darmstadt, Germany) at 4°C for 10 min to counteract the biotinylation effect of the sulfo-NHS-SS-biotin on the cell surface. Cells that were not treated with MESNA were used as a control to examine the total biotinylated EGFR. The cells were then treated with 5 mg/ml iodoacetamide (#I1149; Merck, Darmstadt, Germany) in D-PBS on ice for 5 min to quench free SH groups. Endocytosis was detected by the accumulation of the sulfo-NHS-SS-biotin-labelled cargo proteins within the cells, which were protected from reduction by MESNA. Next, the cells were lysed with lysis buffer and centrifuged, and the supernatant was collected. Except for an aliquot taken to measure the amounts of total cargo proteins, the residual protein lysate was added to streptavidin magnetic beads (#88816; Thermo Scientific, Waltham, MA, USA) and rotated overnight at 4°C. The beads were collected, and the sulfo-NHS-SS-biotin-labeled proteins were eluted in SDS-PAGE sample buffer at 100 °C for 10 min. Finally, the total and internalized biotinylated EGFR was examined by immunoblotting. The internalization ratio was determined by calculating the amount of internalized biotinylated EGFR to total biotinylated EGFR and is expressed as a percentage.

**Ligand-induced EGFR degradation**

Briefly, cells were serum-starved overnight and then treated with 50 µg/mL cycloheximide for 2 hours to block the de novo synthesis of EGFR. Next, the cells were stimulated with 50 ng/ml EGF for 0, 3, 6, 12 hours respectively. Then the incubation was terminated, and the cells were lysed with lysis buffer. Finally, the protein samples were collected and resolved by SDS-PAGE to detect EGFR by immunoblotting.
